# Transcriptomic Profiling of Intracranial Arteries in Adult Patients With Moyamoya Disease Reveals Novel Insights Into Its Pathogenesis

**DOI:** 10.3389/fnmol.2022.881954

**Published:** 2022-05-31

**Authors:** Shuangxiang Xu, Wei Wei, Feiyang Zhang, Tongyu Chen, Lixin Dong, Jichun Shi, Xiaolin Wu, Tingbao Zhang, Zhengwei Li, Jianjian Zhang, Xiang Li, Jincao Chen

**Affiliations:** ^1^Department of Neurosurgery, Zhongnan Hospital, Wuhan University, Wuhan, China; ^2^Brain Research Center, Zhongnan Hospital, Wuhan University, Wuhan, China

**Keywords:** sex difference, RNA sequencing, moyamoya disease, mitochondria, intracranial atherosclerosis

## Abstract

Moyamoya disease (MMD) is a rare, progressively steno-occlusive cerebrovascular disorder of unknown etiology. Here, we revealed the gene expression profile of the intracranial arteries in MMD *via* the RNA-sequencing (RNA-seq). We identified 556 differentially expressed genes (DEGs) for MMD, including 449 and 107 significantly upregulated or downregulated genes. Compared with atherosclerosis-associated intracranial artery stenosis/occlusion (AS-ICASO) controls, upregulated genes were mainly involved in extracellular matrix (ECM) organization, whereas downregulated genes were primarily associated with mitochondrial function and oxidative phosphorylation in MMD. Moreover, we found that a separate sex analysis uncovers more DEGs (*n* = 1.022) compared to an combined sex analysis in MMD. We identified 133 and 439 sex-specific DEGs for men and women in MMD, respectively. About 95.6% of sex-specific DEGs were protein-coding genes and 3% of the genes belonged to long non-coding RNAs (lncRNA). Sex-specific DEGs were observed on all chromosomes, of which 95.49 and 96.59% were autosomal genes in men and women, respectively. These sex-specific DEGs, such as aquaporin-4 (AQP4), superoxide dismutase 3 (SOD3), and nuclear receptor subfamily 4 group A member 1 (NR4A1), may contribute to sex differences in MMD. This transcriptomic study highlighted that ECM and mitochondrial function are the central molecular mechanisms underlying MMD, and revealed sex differences in the gene expression in the intracranial arteries, thereby providing new insights into the pathogenesis of MMD.

## Introduction

Moyamoya disease (MMD) is a rare chronic cerebrovascular disorder characterized by progressive stenosis or occlusion of the distal and intracranial internal carotid arteries (ICAs) and their proximal branches, accompanied by the development of leptomeningeal collaterals, called moyamoya vessels, at the base of the brain (Suzuki and Takaku, 1969; Kuroda and Houkin, 2008). MMD is commonly found worldwide, but it is more prevalent in East Asian countries and that 10–15% of patients with MMD have a family history of the disease (Kuriyama et al., 2008; Kainth et al., 2013; Ahn et al., 2014). Surgical revascularization, including superficial temporal artery (STA) to middle cerebral artery (MCA) bypass, has been proven as an effective treatment for MMD to prevent subsequent stroke and rebleeding (Miyamoto et al., 2014).

Although the etiology and pathogenesis of MMD are still unknown, genetic factors have been implicated in the development of this disease. This idea is supported by the differences in the incidence of MMD in different ethnic populations mentioned above. In addition, numerous genome-wide associated studies revealed that the ring finger protein 213 (RNF213) p.R4810K mutation was an important susceptibility gene for MMD through whole blood samples (Kamada et al., 2011; Liu et al., 2011; Miyawaki et al., 2012; Duan et al., 2018; Cheng et al., 2019). However, this mutation showed a marked variation in frequency among ethnic populations [e.g., about 90% in Japanese, 80% in Korean, only 20% in Chinese (Liu et al., 2011; Cheng et al., 2019); and no detectability in Caucasian patients with MMD (Guey et al., 2017)]. This suggested that the mutation of this gene may not be solely responsible for MMD and that other causes of susceptibility for MMD may exist. Recently, a previous study has identified hundreds of differentially expressed genes (DEGs) in the peripheral blood of MMD and healthy subjects through RNA-sequencing (RNA-seq) (Peng et al., 2019). This result indicates that molecular mechanism based on genome is critical for understanding the etiology and pathogenesis of MMD.

The samples investigated in genome-wide or RNA-seq studies were mainly obtained from patients' peripheral blood, which may not reflect the real pathological changes of blood vessels in MMD. Because it is difficult to extract sufficient RNA or proteins from the microsamples of MCA that can be obtained during surgery, studies to elucidate the genome-wide molecular characteristics of the intracranial arteries have rarely been reported. Excitingly, two recent studies from Japan reported a transcriptome-wide analysis of intracranial arteries in patients with MMD *via* an RNA microarray (Okami et al., 2015; Kanamori et al., 2021). However, the microarray analysis has several limitations, including its reliance on known genome sequence, high background noise, a narrow dynamic range of detection, and a limited ability to distinguish different isoforms and allelic expression, compared with high-throughput sequencing (Wang et al., 2009).

Importantly, our previous study and other studies have proven that the cortical segments of MCA specimens from patients with MMD have significantly thinner media and thicker intima than MCAs extracted from atherosclerosis-associated intracranial artery stenosis/occlusion (AS-ICASO) (Takagi et al., 2006, 2016; Zhang et al., 2016). Moreover, MMD and AS-ICASO have similar pathological progression, including chronic and slow progression of intracranial artery stenosis/occlusion. Therefore, the MCA specimen from AS-ICASO could serve as an ideal control to understand the actual pathogenesis of MMD.

In this study, tiny pieces of the cortical segment of the MCA were harvested from 16 adult patients with MMD and five patients with matched AS-ICASO during their STA-MCA bypass surgeries, and then high-throughput RNA-seq was performed to detect DEGs. To our knowledge, this study is the first time to report genome-wide transcriptomic profiling of the intracranial artery specimens from patients with MMD *via* the RNA-seq.

## Methods and Materials

### Patients and Specimen Preparation

**Figure 1:**
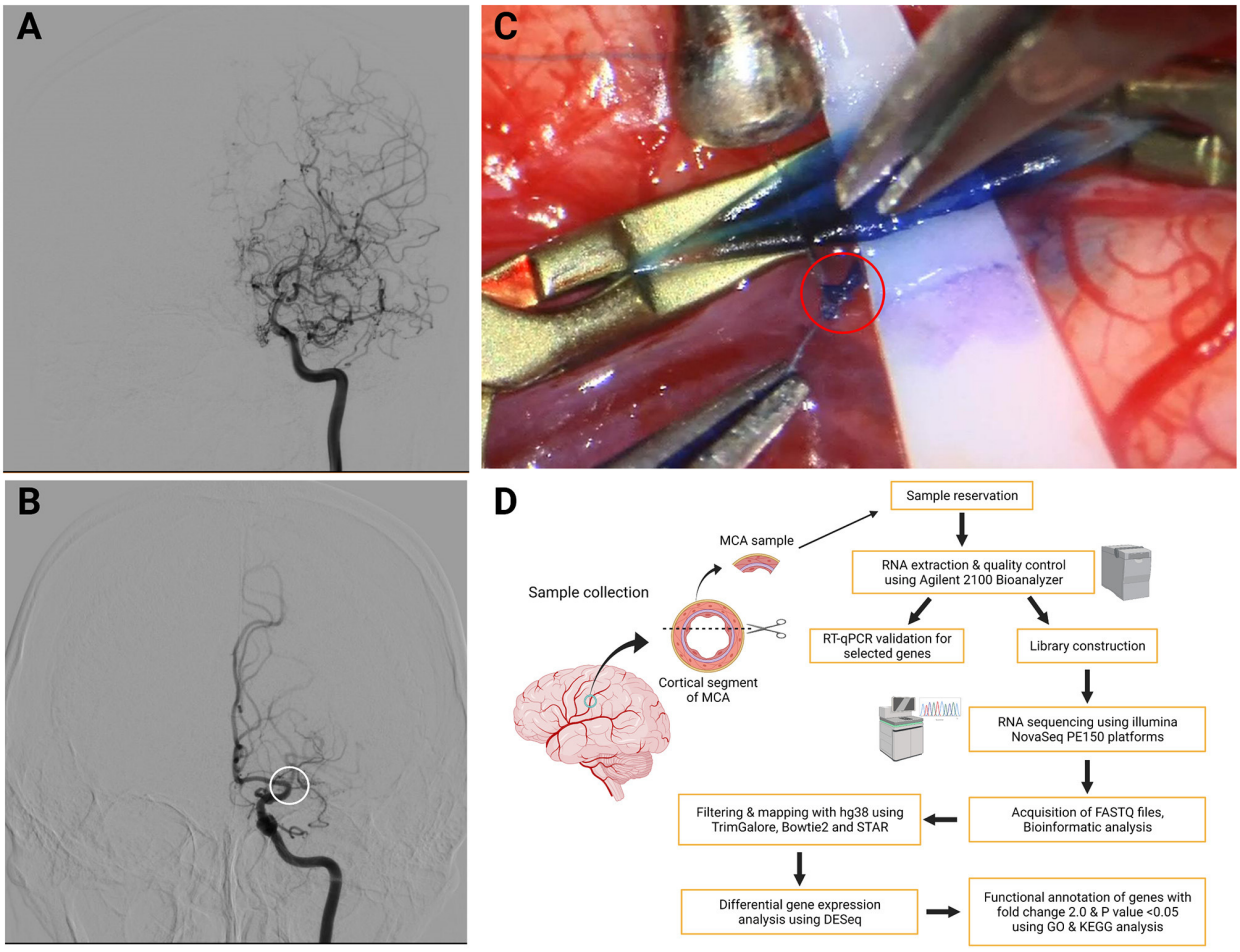
Typical digital subtraction angiography (DSA) images for diagnosis, intraoperative image of obtaining middle cerebral artery (MCA) specimens, and workflow diagram of this study. **(A)** Typical image of Moyamoya disease (MMD). It shows stenosis-occlusion in the left terminal portion of the internal carotid arteries (ICAs) and the proximal segment of anterior cerebral artery (ACA) and MCA accompanied by moyamoya vessels. **(B)** Typical image of atherosclerosis-associated intracranial artery stenosis/occlusion (AS-ICASO). It shows the left M1 segment of MCA occlusion (white circle) without moyamoya vessels. **(C)** Arteriotomy was performed cautiously with microscissors, and a tiny piece of specimen was harvested from the artery in the red circle. **(D)** Workflow diagram of this study. *Created with BioRender.com*.

In total, 16 adult patients with MMD and five with AS-ICASO in the Chinese Han population were enrolled in this study. Detailed baseline data and clinical characteristics of all patients are summarized in Supplementary Table 1. The diagnostic criteria for MMD were based on the guidelines published in 2012 by the Research Committee on the Pathology Treatment of Spontaneous Occlusion of the Circle of Willis (2012) of the Ministry of Health and Welfare, Japan. Digital subtraction angiography (DSA) was applied to diagnose MMD and AS-ICASO (Figures 1A,B). A computer tomography (CT) or magnetic resonance imaging (MRI) scan was also performed for each patient to determine the clinical manifestation of patients with MMD and controls. All MMD and AS-ICASO cases were treated by standard STA-MCA bypass surgery, and the cortical segment of MCA specimens was collected intraoperatively. Surgically resected tissues were frozen in liquid nitrogen as soon as possible and then stored at −80°C until the subsequent experiment.

All vascular specimens were obtained by applying the technique described in our previous study (Figure 1C) (Zhang et al., 2016). The research protocol was approved by the Institutional Ethics Committee of Zhongnan Hospital, Wuhan University based on the guidelines of the 1964 Declaration of Helsinki (No. 2021028). Informed consent was obtained from all individuals prior to their enrollment. The workflow diagram of this study is shown in Figure 1D.

### RNA Extraction

Total RNA was extracted from the MCA samples using RNAiso Plus reagent (Takara, Otsu, Japan) following the manufacturer's protocol. Harvested RNA was quantified with the Qubit® 4.0 Fluorometer (Thermo Scientific, USA). To evaluate the purity and integrity of RNA, Agilent 2100 Bioanalyzer (Agilent Technologies, Santa Clara, CA, USA) and an RNA 6000 Pico Chip kit (Agilent Technologies) were used.

### RNA Library Construction and RNA-Seq

In total, 10 ng of RNAs was used to construct the RNA library using the SMARTer® Stranded Total RNA-Seq kit v2—Pico Input Mammalian (Takara, Mountain View, CA, USA) according to the manufacturer's protocol, which contained a series of procedures, including first-strand cDNA synthesis, addition of Illumina adapters and barcodes, library purification, ribosomal cDNA depletion, PCR amplification, library purification, and quality control. Library concentration was detected using the Qubit® 4.0 Fluorometer (Thermo Scientific), and the quality control was determined using an Agilent 2100 Bioanalyzer with a DNA 1000 Chip kit (Agilent Technologies). Subsequently, the RNA-seq was carried out using Illumina NovaSeq PE150 platforms at Beijing Annoroad Biotechnology Co. Ltd. (Beijing, China).

### Quantitative Polymerase Chain Reaction

To measure gene expression levels, quantitative polymerase chain reaction (qPCR) was conducted. After the extraction of total RNA, as mentioned above, reverse transcription was performed using the HiScript III 1st Strand cDNA Synthesis kit (+gDNA wiper) (Vazyme, Nanjing, China), and thus cDNA amplification using the ChamQ Universal SYBR qPCR Master Mix (Vazyme, Nanjing, China). Gene expression was determined by the comparative Ct method and normalized to that of GAPDH. All primers have been synthesized by Sangon Biotech (Shanghai, China) and are listed in Supplementary Table 2.

### Identification of an RNF 213 p.R4810K Variant

Genomic DNA was isolated from 200 μl of peripheral blood using the FastPure Blood DNA Isolation Mini kit (Vazyme, Nanjing, China). RNF213 was amplified *via* PCR using a Phanta Max Super-Fidelity DNA Polymerase (Vazyme, Nanjing, China) according to the manufacturer's protocol. Primer sequences have been synthesized by Sangon Biotech (Shanghai, China) and are shown in Supplementary Table 2. After agarose gel electrophoresis, the PCR product was purified using a SanPrep Column DNA Gel Extraction kit (Sangon Biotech, Shanghai, China). The existence of the p.R4810K variant of RNF213 (rs112735431) was identified by Sanger sequencing with the Big Dye Terminator version 1.1 kit (ThermoFisher) and an ABI 3730XL Genetic Analyzer (Applied Biosystems, USA).

### Qualification and Quantification of mRNA

Illumina high-throughput sequencing data were dealt with bcl2fastq and converted into sequenced reads (raw reads). Fastq files were filtered by TrimGalore (https://github.com/FelixKrueger/TrimGalore) with the criteria of Ph red quality score of 30 and a minimum length of 25. Bowtie2 (http://bowtie-bio.sourceforge.net/bowtie2/index.shtml) was applied to filtrate rRNA reads by aligning against the human rRNA fasta file from NCBI and to build an index from the genome annotation file hg38, which was downloaded from Ensembl (https://www.ensembl.org/index.html). Unmapped reads were then mapped to hg38 index by STAR (Dobin et al., 2013). We then used samtools (version:1.8) (http://www.htslib.org/) to convert sam files to bam files along with sorting, which was dealt with FeatureCounts (Liao et al., 2014) to create a gene expression matrix with parameters “-t exon -g gene_id.”

### DEGs of MMD and AS-ICASO Groups

The expression matrix was imported to R (version:4.0.4) for a downstream analysis. DESeq2 (1.30.1) (Love et al., 2014) was applied to identify DEGs between the MMD group and AS-ICASO group. In addition, the values of *p* were calculated using the Wald test, which were corrected with the Benjamini and Hochberg method for multiple testing by default of DESeq2. Significant genes were defined with a minimum log2 fold change of 1 and the value of *p* < 0.05. We ran a principal component analysis (PCA) reduction analysis on all DEGs. Clustering for patients is calculated based on using the hclust function. ClusterProfiler (verision:3.18.1) (Wu et al., 2021) is applied for both Gene Ontology (GO) and Kyoto Encyclopedia of Genes and Genomes (KEGG) analyses. The R packages stringr, dyplr, ggplot2, ComplexHeatmap, circlize, factoextra, psych, gridExtra, AnnotationDbi, factoextra, and psych were employed in the workflow of data processing and visualization.

### Sex-Specific MMD-Related Genes

Sex-specific MMD-related genes were defined as genes related not only to the progression of MMD, but also differing in men and women with MMD. Thus, we firstly found MMD progression-related genes in men and women. Using the same criteria for significant genes as stated above, 424 genes and 598 genes in men and women were found as Sex-respective Genes, which indicated a significant expression pattern shift within each group but might not vary between all groups. For this purpose, we further survey the sex-specific genes, which have a significant alteration in the expression, we found DEGs between men and women with MMD at the same threshold, followed by intersecting such genes with sex-respective Genes. In total, 133 sex-specific Genes were found in men and 439 in women.

### Statistical Analysis

Demographic variables were represented as the mean ± standard deviation (SD) between the MMD group and the control group unless otherwise specified. Statistical analyses were performed using an unpaired student's *t*-test for continuous variables and the Fisher's exact test for categorical variables. *p* < 0.05 was considered as statistically significant. Statistical analysis was performed using GraphPad Prism version 9.3.0.

## Results

### Demographic and Clinical Characteristics of Participants

The demographic and clinical characteristics of participants are summarized in Table 1. The age was 54.94 ± 5.53 and 59.60 ± 10.64 years in the MMD and control group, respectively. The initial manifestations of patients with MMD included intraventricular hemorrhage (IVH), intracerebral hemorrhage (ICH), subarachnoid hemorrhage (SAH), transient ischemic attack (TIA), and cerebral infarction (CI). Of the 16 patients with MMD, over half (56.25%) presented with a symptom of TIA or CI. In the AS-ICASO cases, four patients presented with CI and one patient suffered TIA. There were no significant difference in age, sex, and clinical manifestations between MMD and AS-ICASO. For medical history, atherosclerosis-associated diseases, such as hypertension, diabetes mellitus, and hyperlipidemia, were significantly higher in the AS-ICASO group than in the MMD group (*p* = 0.035). The RNF213 p.R4810K mutation was not detected in either the MMD or the AS-ICASO group. This strongly suggested that the RNF213 p.R4810K mutation was not the main susceptible cause for the Chinese MMD population. The duration between the last symptom and surgery in the MMD group and control group is 120.6 ± 53.5 and 104.2 ± 4.4 days, respectively (Supplementary Table 1). There is no significant difference between these two groups.

**Table 1:** Summary of the demographic and clinical characteristics of all participants.

**Items**	**MMD (*n* = 16)**	**AS-ICASO (*n* = 5)**	***p***
Age (mean ± SD)	54.94 ± 5.53	59.60 ± 10.64	0.204
Sex (male/female)	8/8	3/2	0.999
Family history of MMD	0	0	0.999
**Initial symptom**
ICH	4	0	0.532
IVH	2	0	0.999
SAH	1	0	0.999
CI	7	4	0.311
TIA	2	1	0.999
Subtotal (hemorrhage/	7/9	0/5	0.123
ischemia)
**Medical history**
Hypertension	4	4^*^	0.047
Diabetes mellitus	2	2	0.228
Hyperlipidemia	0	1	0.238
Subtotal	6	5	0.035
Mutation in RNF213	0	0	0.999

*Values represent the number of patients unless indicated otherwise. Statistical analyses were performed using an unpaired student's t-test for continuous variables and the Fisher's exact test for categorical variables. ICH, intracerebral hemorrhage; IVH, intraventricular hemorrhage; SAH, subarachnoid hemorrhage; TIA, transient ischemic attack; CI, cerebral infarction*.

* *In the atherosclerosis-associated intracranial artery stenosis/occlusion (AS-ICASO) group, two subjects have both hypertension and diabetes mellitus*.

### Genome-Wide Transcriptomic Profiling in MCA Specimens of Patients With MMD

**Figure 2:**
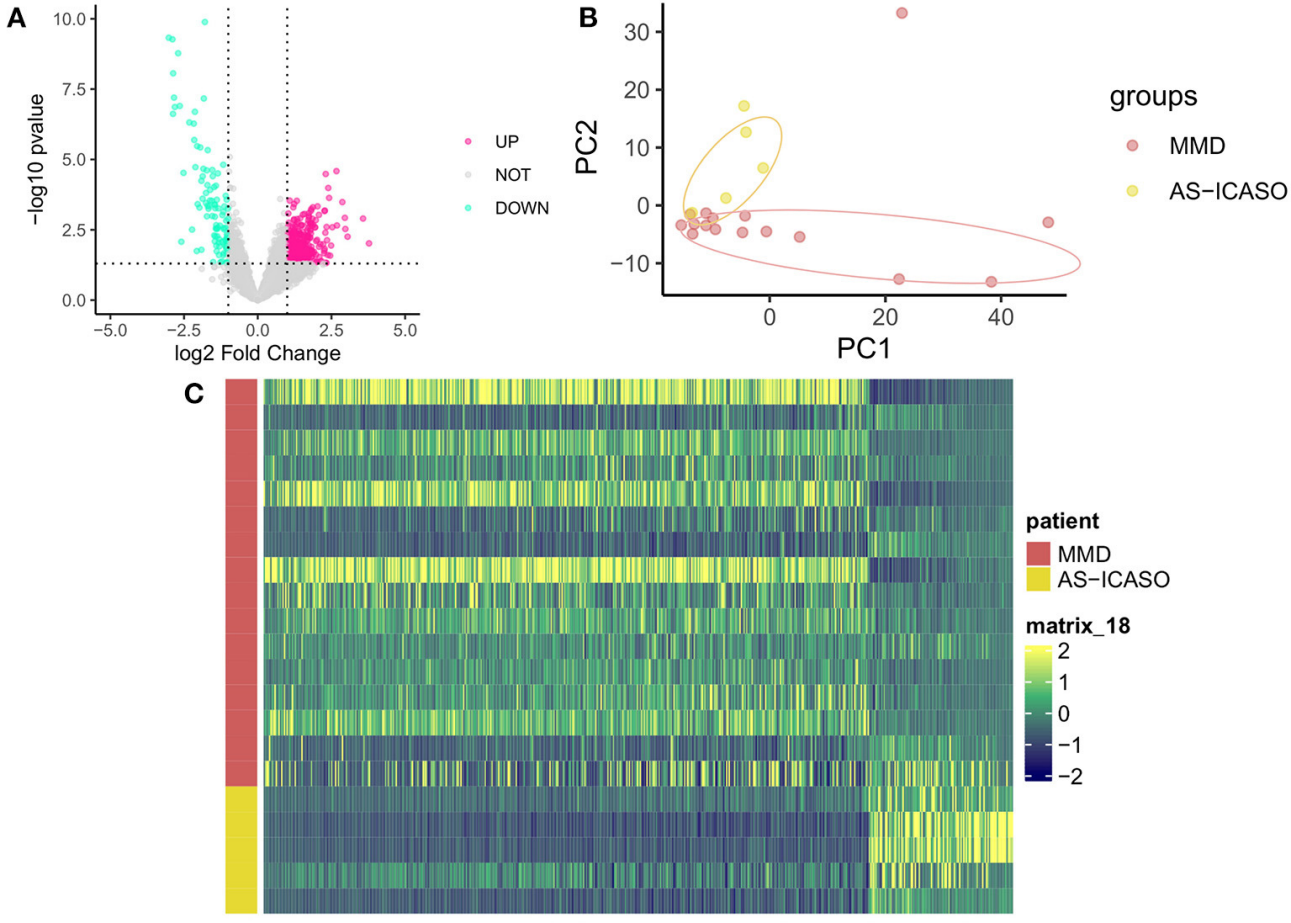
Dysregulated genes in MCA specimens from MMD and AS-ICASO controls. **(A)** A volcano plot to visualize the DEGs between MMD and AS-ICASO controls. *Magenta dots* represent significantly (*p* < 0.05 and fold change >2) upregulated genes (*n* = 449), and *indigo dots* represent significantly downregulated genes (*n* = 107). *Gary dots* represent the genes that are not differentially expressed. **(B)** A principal component analysis (PCA) biplot for PC1 and PC2, performed using all of the normalized values obtained from the sequencing data, shows two separate clusters for MMD and AS-ICASO controls. *Dark red dots* represent patients with MMD, *yellow dots* represent participants with AS-ICASO. **(C)** Heatmap shows a distinct expression pattern of DEGs between MMD and AS-ICASO controls. The color key at the right indicates a relative gene expression. Yellow and green colors represent higher and lower gene expression levels, respectively.

To further compare the differences in the gene expression pattern in MCA between MMD and AS-ICASO, we performed the RNA-seq on MCA specimens derived from patients with MMD or AS-ICASO using the SMARTer-seq, which allowed us to explore genome-wide transcriptomic profiling from low-input samples. As a result, we identified 556 DEGs between the MMD and AS-ICASO group, in which 449 and 107 genes were significantly upregulated or downregulated in MMD (Figure 2A). Intriguingly, PCA using the MCA sequencing data of participants showed obviously distinct clusters for the MMD and AS-ICASO group (Figure 2B). An heatmap analysis based on the gene expression of the 556 DEGs demonstrated that MMD and AS-ICASO had a distinct gene expression pattern (Figure 2C). Taken together, the global analysis of the gene expression in MCA specimens in MMD reveals previously unknown differential expression for a multitude of genes. Furthermore, according to their fold change, we ranked the 556 DEGs and found that many of the top upregulated or downregulated DEGs were mainly related to mitochondrial function, extracellular matrix (ECM), angiogenesis, immune, and inflammatory response (Supplementary Table 3). These results suggested that MMD, an uncommon cerebrovascular disorder, could display significant gene dysregulation in the intracranial arteries.

### qPCR Validation of Selected Genes

**Figure 3:**
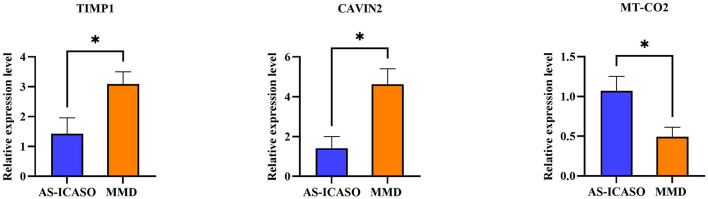
Quantitative polymerase chain reaction (qPCR) validation of the selected genes. The validation of the RNA-sequencing (RNA-seq) results using qPCR in independent samples including nine patients with MMD and six AS-ICASO controls. The relative expression level of mRNA is shown. Expression values are first normalized to glyceraldehyde 3-phosphate dehydrogenase (GAPDH; internal control) and then plotted relative to corresponding controls that are set as one for each gene. Error bar represents mean ± standard error of the mean (SEM). Unpaired student's *t*-test is used to calculate significant differences. *p < 0.05.

Because of a number of MCA specimens, we have to select several relative genes for qPCR validation using another independent sample from MMD and AS-ICASO. We selected the genes according to the following criteria: (1) the genes showed significant differential expression in RNA-seq analysis; (2) gene functions were expected to contribute to the pathophysiology of MMD. Finally, metallopeptidase inhibitor 1 (TIMP1), caveolae-associated protein 2 (CAVIN2), and mitochondrially encoded cytochrome c oxidase II (MT-CO2) were chosen for validation. According to the results of qPCR, the expression of these transcripts between the MMD and control group has demonstrated a consistent distribution in the gene expression as observed in the RNA-seq (Figure 3).

### GO and KEGG Pathway Enrichment Analyses

**Figure 4:**
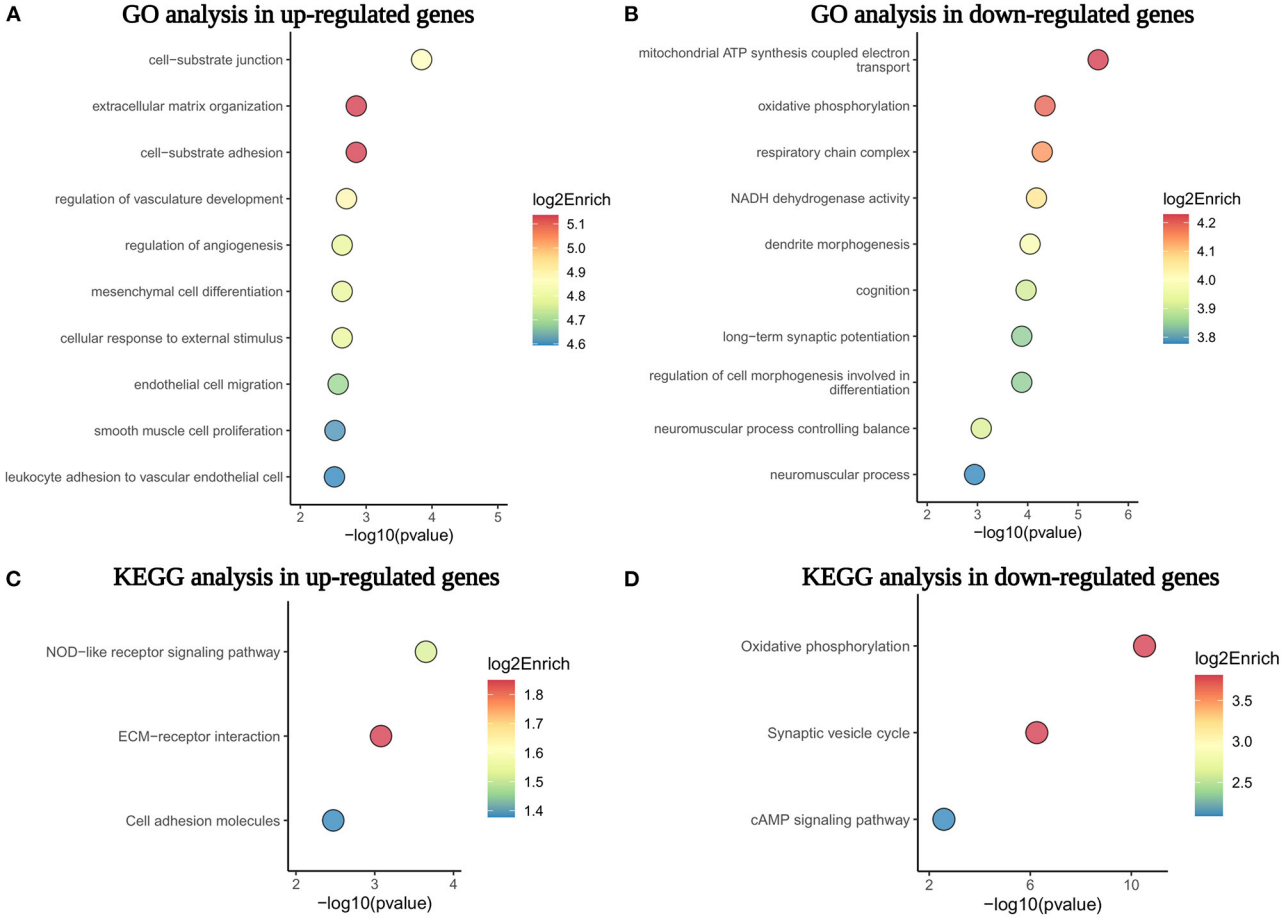
The GO and KEGG pathway enrichment analysis of dysregulated genes in the MCA of patients with MMD. **(A,B)** Showed the top 10 GO biological processes enriched in upregulated and downregulated genes, respectively. **(C,D)** Displayed the top three KEGG pathways overrepresented in upregulated and downregulated genes. GO, Gene Ontology; KEGG, Kyoto Encyclopedia of Genes and Genomes; ECM, extracellular matrix.

To further understand the biological processes and pathways of DEGs, we performed the GO and KEGG pathway enrichment analysis (Bonetta, 2004). As a result, the GO analysis demonstrated that numerous genes upregulated in MMD were related to cell-substrate junction, ECM organization, cell-substrate adhesion, the regulation of vasculature development, and the regulation of angiogenesis (Figure 4A). In contrast, the downregulated genes in the MMD group were mainly involved in mitochondrial ATP synthesis-coupled electron transport, the respiratory chain complex, oxidative phosphorylation, a neuromuscular process controlling balance, and dendrite development (Figure 4B). The KEGG pathway enrichment analysis uncovered that NOD-like receptor signaling pathway, ECM–receptor interaction, and cell adhesion molecules were enriched in upregulated genes, on the contrary, oxidative phosphorylation, synaptic vesicle cycle, and cAMP signaling pathway were overrepresented in downregulated genes in patients with MMD (Figures 4C,D). Therefore, our results suggested that these enriched biological processes or pathways, such as ECM organization, mitochondrial function, and oxidative phosphorylation, may be closely related to the complex pathogenesis of MMD.

### Sex Differences Between Adult Men and Women Patients With MMD

Previous studies have reported the presence of sex differences among men and women with MMD, mainly in incidence and clinical manifestation based on epidemiological results (Baba et al., 2008; Kuriyama et al., 2008; Kainth et al., 2013; Ahn et al., 2014). As sex differences were known to exist in men and women with MMD, we further analyzed gene expression, in MCA, between men and women patients with MMD (*n* = 8/8). There were no significant differences in age, clinical manifestations, and medical history between men and women with MMD (Supplementary Table 1).

**Figure 5:**
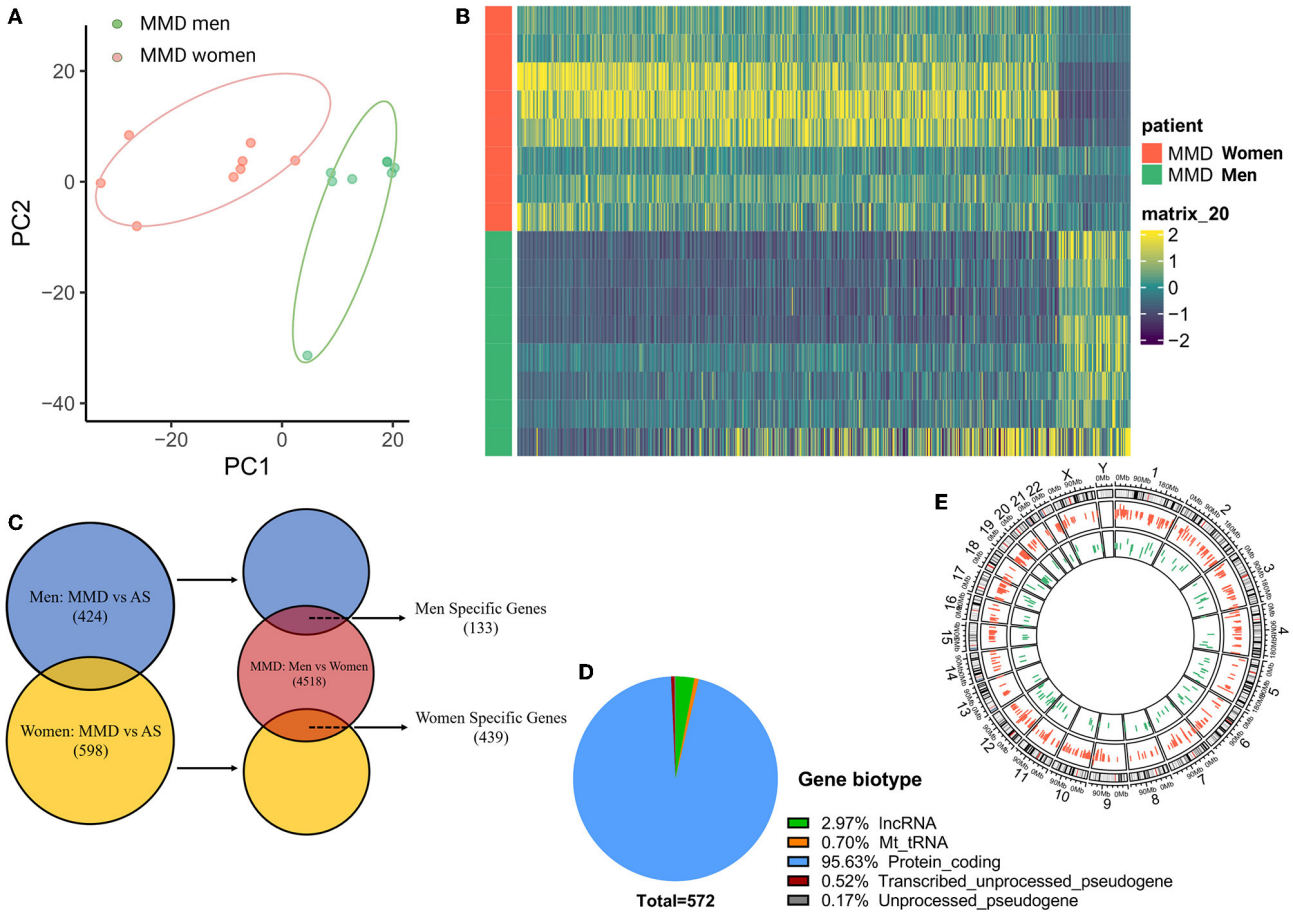
Sex differences between adult men and women patients with MMD. **(A)** PCA analysis, performed using all of the normalized values obtained from the sequencing data, showed two separate clusters for men and women with MMD. *Green dots* represent men patients with MMD, *red dots* represent women participants with MMD. **(B)** Heatmap showed a distinct gender expression pattern of DEGs in MMD. The color key at the right indicates a relative gene expression. Yellow and green colors represent higher and lower gene expression levels, respectively. **(C)** Venn plot of sex-specific DEGs showed specific genes in men and women patients with MMD. Blue color represents men with MMD vs. men with AS-ICASO, yellow color represents women with MMD vs. women with AS-ICASO, and orange color represents men with MMD vs. women with MMD. **(D)** Gene biotype of all sex-specific genes. **(E)** Chromosome distribution of sex-specific genes shows that 95.49 and 96.59% of the genes are autosomal genes in men and women, respectively. *Green bar* represents men patients with MMD, *red bar* represents women patients.

Interestingly, in our transcriptomic study, we observed the existence of sex differences in the gene expression in men and women with MMD. According to the PCA analysis, men and women with MMD were completely categorized into two separate clusters (Figure 5A). There was a distinct gene expression pattern according to sex in MMD (Figure 5B). In the MMD group, we identified 4,518 sex DEGs when sex was analyzed separately. To further distinguish sex DEGs, which were related to the progression of MMD, we compared men with MMD vs. AS-ICASO and women with MMD vs. AS-ICASO. Thus, we identified 424 DEGs in men with MMD and 598 DEGs in women with MMD (Figure 5C). MMD-related sex-specific genes were defined as genes related not only to the progression of MMD, but also differing in men and women with MMD. As a result, we identified 133 and 439 sex-specific DEGs in men and women with MMD, respectively (Figure 5C), of which most of the sex-specific DEGs were newly observed dysregulations in the intracranial arteries of patients with MMD. According to the gene biotype, the majority of sex-specific DEGs (about 95.6%) were protein-coding genes and about 3% of the genes belonged to long non-coding RNAs (lncRNA) (Figure 5D). Sex-specific DEGs were observed on all chromosomes, of which 95.49 and 96.59% were autosomal genes in men and women, respectively (Figure 5E).

To extend our understanding of sex-specific expressed genes, we examined genes that are upexpressed or downexpressed between men and women. In men with MMD, 50 and 83 sex-specific DEGs were upregulated or downregulated, respectively. Of these, the top five sex-specific genes in our analysis with a significantly higher expression were aquaporin-4 (AQP4), FUT9, KCNH7, CADM2, and LSAMP. On the other hand, the top five sex-specific genes with a significantly lower expression were ATP1A2, F3, LGI4, METTL7A, and DST (Table 2).

**Table 2:** Top 10 upregulated or downregulated sex-specific differentially expressed genes (DEGs) in middle cerebral artery (MCA) samples in men patients with Moyamoya disease (MMD).

**Gene symbol**	**Ensemble ID**	**Fold change**	***p***
**Upregulated genes**
AQP4	ENSG00000171885	5.2	5.8E-05
FUT9	ENSG00000172461	4.7	3.8E-04
KCNH7	ENSG00000184611	4.5	2.0E-05
CADM2	ENSG00000175161	3.9	2.0E-04
LSAMP	ENSG00000185565	3.8	1.5E-05
PPM1E	ENSG00000175175	3.5	7.0E-05
NEFL	ENSG00000277586	3.5	2.1E-03
KIF14	ENSG00000118193	3.4	1.0E-04
LOC105379109	ENSG00000251574	3.1	1.1E-04
CENPE	ENSG00000138778	2.9	1.0E-05
**Downregulated genes**
ATP1A2	ENSG00000018625	6.4	7.0E-07
F3	ENSG00000117525	6.2	2.5E-08
LGI4	ENSG00000153902	4.3	1.6E-07
METTL7A	ENSG00000185432	4.1	6.0E-05
DST	ENSG00000151914	3.6	1.5E-05
CPE	ENSG00000109472	3.5	1.4E-02
SRRM2	ENSG00000167978	3.5	1.0E-03
NARS1	ENSG00000134440	3.4	1.7E-05
CCDC124	ENSG00000007080	3.3	3.3E-04
RAD23A	ENSG00000179262	3.3	4.5E-04

For women with MMD, 422 sex-specific DEGs were upregulated, whereas only 17 were downregulated. The top five sex-specific genes with a significantly higher expression were superoxide dismutase 3 (SOD3), nuclear receptor subfamily 4 group A member 1 (NR4A1), PPDPF, AEBP1, and MYADM. On the other hand, CTNND2, CAMK2N1, KIF5A, NRXN1, and OLFM1 were the top five genes that were significantly decreased (Table 3).

**Table 3:** Top 10 upregulated or downregulated sex-specific DEGs in MCA samples in women patients with MMD.

**Gene symbol**	**Ensemble ID**	**Fold change**	***p***
**Upregulated genes**
SOD3	ENSG00000109610	18.0	1.03E-13
NR4A1	ENSG00000123358	15.9	8.16E-10
PPDPF	ENSG00000125534	14.7	5.57E-15
AEBP1	ENSG00000106624	13.2	3.02E-12
MYADM	ENSG00000179820	12.6	3.97E-13
PNRC1	ENSG00000146278	12.6	3.87E-12
AHNAK	ENSG00000124942	12.5	1.99E-08
COL1A2	ENSG00000164692	12.2	5.75E-15
IER2	ENSG00000160888	12.0	1.44E-11
IGFBP5	ENSG00000115461	11.8	1.30E-10
**Downregulated genes**
CTNND2	ENSG00000169862	3.2	1.0E-03
CAMK2N1	ENSG00000162545	3.0	2.5E-02
KIF5A	ENSG00000155980	3.0	3.1E-06
NRXN1	ENSG00000179915	2.9	6.8E-05
OLFM1	ENSG00000130558	2.9	1.6E-03
CAMK2A	ENSG00000070808	2.7	1.3E-03
CNKSR2	ENSG00000149970	2.5	6.1E-05
SCN8A	ENSG00000196876	2.5	9.1E-06
CAMK2B	ENSG00000058404	2.5	5.6E-03
SRGAP3	ENSG00000196220	2.3	1.1E-04

**Figure 6:**
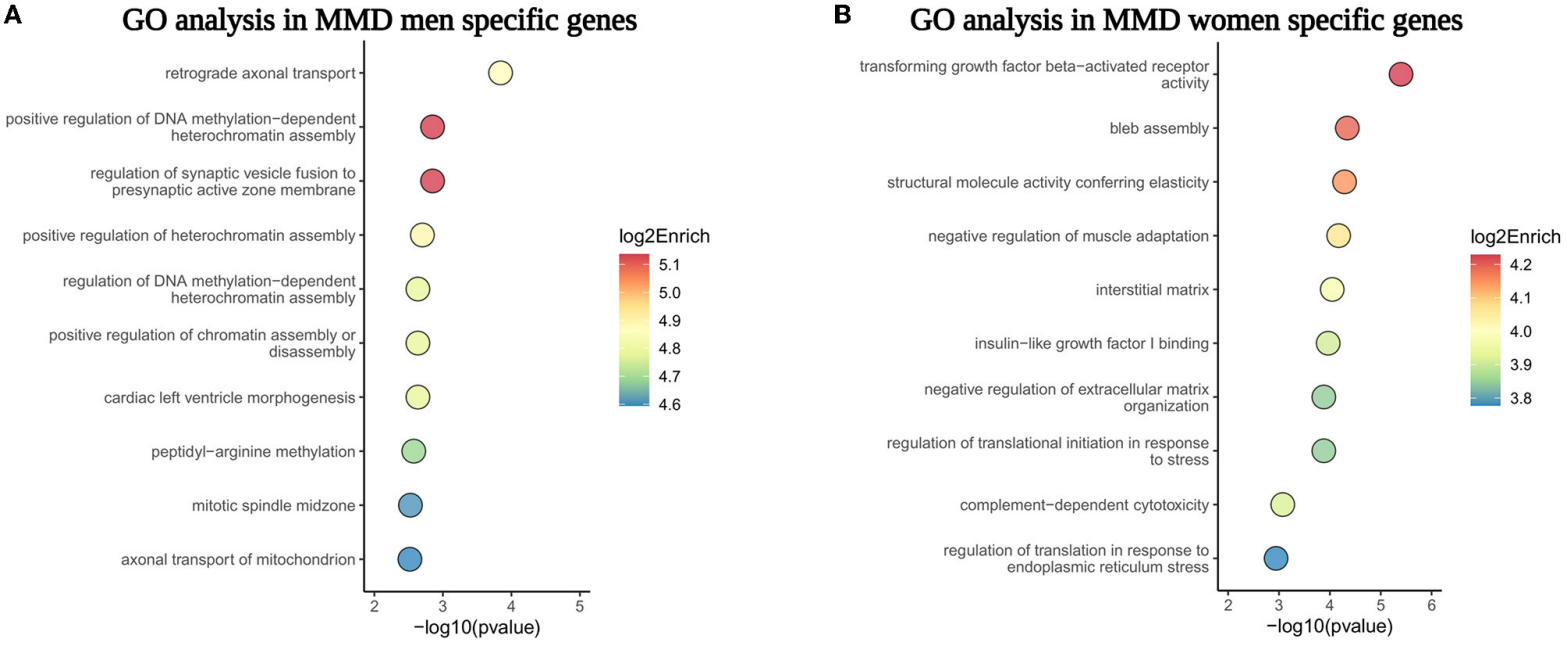
The GO pathway enrichment analysis of sex-specific genes in MCA of men and women patients with MMD. **(A,B)** Showed the top 10 GO biological processes enriched in male and female sex-specific genes, respectively.

The pathway enrichment analysis of sex-specific DEGs showed that retrograde axonal transport and the regulation of DNA methylation were predominant bioprocesses in men with MMD, whereas transforming growth factor-β- (TGFβ-) activated receptor activity and bleb assembly were the main biological processes in women with MMD (Figures 6A,B). These sex differences in the gene expression indicate that the pathophysiology of MMD may be different for men and women.

### Differences Between Ischemic and Hemorrhagic MMD

Taking it into account that different types of MMD manifestation, such as ischemia and hemorrhage, may have effects on gene expression, we also analyzed the gene expression profile between ischemic and hemorrhagic MMD. According to our analysis, we identified a total of 633 hemorrhagic-ischemic DEGs (HI-DEGs) using the same criterion, as mentioned in the methods (Supplementary Table 4). However, there were no clear differences between the hemorrhagic group and the ischemic group, as shown in the heatmap and PCA (Supplementary Figures 1, 2). This result showed that variants in gene expression randomly cross the transcriptome rather than affecting certain genes, and may suggest that the pathology between ischemic and hemorrhagic MMD is very similar.

## Discussion

Moyamoya disease is characterized by progressive stenosis/occlusion in the terminal portion of ICA and main vascular branches in the circle of Willis (Kuroda and Houkin, 2008). Moreover, moyamoya-like stenosis changes almost do not occur in the external carotid system or intra-abdominal arteries in patients with MMD (Komiyama et al., 2000; Togao et al., 2004). These studies indicate that the core pathogenesis of MMD is restricted to the intracranial arteries. Therefore, investigating the molecular characteristics of intracranial arteries is vital to understand the pathogenesis of MMD. As such, we believe that using intracranial artery samples of MMD to perform genome-wide analysis is better than peripheral blood samples, which may not be suitable for studying the actual molecular mechanism of MMD. To our knowledge, this study is the first time to perform transcriptomic profiling of the intracranial arteries of patients with MMD *via* the RNA-seq.

As a result, we identified 449 and 107 genes significantly upregulated or downregulated in MMD, implying a distinct gene expression profile of the intracranial arteries between MMD and AS-ICASO control. In addition, we observed the presence of sex differences in the gene expression of the intracranial arteries in men and women with MMD. Although we identified 633 DEGs between ischemic and hemorrhagic patients with MMD, hemorrhagic patients with MMD and ischemic patients with MMD could not be completely separated from each other either in a heatmap or in the PCA plot. It is well-known that ischemic patients with MMD may progress to hemorrhage if not treated in time while a hemorrhagic patient with MMD could also show insufficient cerebral perfusion.

On the other hand, the selection of controls is different from that of a previous study, in which patients with intracranial aneurysm or epilepsy are selected as controls (Kanamori et al., 2021). As an ischemic cerebrovascular disease, stenosis caused by MMD and AS-ICASO share a series of similarities in clinical presentations and pathological changes. For instance, ischemic cerebrovascular events are the most common manifestations in both diseases, whereas hemorrhagic stroke may present in patients with MMD due to the rupture of fragile moyamoya vessels or microaneurysms (Kuroda and Houkin, 2008). In terms of histopathological changes, similar to MMD, AS-ICASO causes stenosis with intimal expansion and intimal smooth muscle cell hyperplasia. However, AS-ICASO is marked by a characteristic inflammatory infiltration and lipid accumulation that do not typically occur in MMD (Fukui et al., 2000; Lin et al., 2012). Interestingly, studies have found that moyamoya-like stenosis changes almost do not occur in the external carotid system or intra-abdominal arteries in patients with MMD (Komiyama et al., 2000; Togao et al., 2004). The restriction of MMD stenosis to such a limited and specific anatomic region remains one of the most fundamental questions surrounding its pathology, as other forms of medium to large artery stenosis, such as atherosclerosis, are typically far more widespread throughout the arterial tree (Fox et al., 2021). In terms of molecular mechanisms, many angiogenic factors, such as vascular endothelial growth factor (VEGF), TGFβ, and hypoxia inducible factor 1α (HIF1α), and the RNF 213 variant, are not only associated with MMD but also connected with AS-ICASO compared with healthy controls (Burke et al., 2009; Lin and Sheng, 2018; Shoeibi et al., 2018). Generally, MMD and AS-ICASO are two different disease entities with distinctive pathogenesis and therapeutic strategies, but both share a series of similarities and differences in clinical presentations, pathological changes, and molecular mechanisms. Given this, the contrast of MMD with AS-ICASO could highlight the central questions underlying its pathogenesis.

Based on the GO and KEGG pathway analysis, genes involved in ECM organization were upregulated, while genes involved in mitochondrial function and oxidative phosphorylation were downregulated in MMD vs. AS-ICASO. The ECM organization was identified as enriched in MMD through the GO analysis, and this result was consistent with a recent study that performed the RNA-seq using peripheral blood in MMD (Peng et al., 2019). Regarding the KEGG pathway enrichment analysis, the analysis illustrated that ECM–receptor interaction was the pathway that is overrepresented in patients with MMD. Similarly, a previous study reported that the downregulation of ECM–receptor-related genes may be associated with impaired angiogenic activity in ECs derived from iPSCs in patients with MMD (Hamauchi et al., 2016). Timely regulation of ECM is an important feature of tissue development, morphogenesis, repair, and remodeling. It is mainly regulated by the balance between matrix metalloproteinases (MMPs) and tissue inhibitor of metalloproteases (TIMPs) (Nagase et al., 2006). Accumulating evidence has proven that the disrupted balance between MMPs and TIMPs plays a potential role in the pathogenesis of MMD (Kang et al., 2010; Bang et al., 2016b). Similarly, we found that patients with MMD exhibited an obviously higher expression of MMP2 (*p* = 0.001) and TIMP1 (*p* = 0.04) in MCA specimens than that of controls in the present study. Given that peri-endothelial ECM plays an important role in protection, cell adhesion and migration, dysfunctional peri-endothelial ECM in MMD may contribute to endothelial vulnerability to wall shear stress. As a result, invasive endothelial progenitor cells repairing endothelial injury would produce excess hyaluronan and CS in the intima, and cause vascular stenosis (Matsuo et al., 2021). A previous study has found a different pattern of vascular remodeling in MMD and AS-ICASO. During the progression of luminal stenosis, expansive vascular remodeling occurs in AS-ICASO accompanied by an increase in vessel outer diameter (Xu et al., 2010), in contrast to a decrease in the outer diameter that occurs in MMD (Kaku et al., 2012). These results supported the hypothesis that ECM may be involved in vascular stenosis or occlusion and the formation of an abnormal vascular network in patients with MMD.

In our study, the gene expression of CAVIN2 and caveolin 1 (CAV1) was significantly upregulated (*p* = 0.01, *p* = 0.02, respectively) in MCA samples of patients with MMD. As a principal scaffolding protein component of caveolae, CAVIN2 and CAV1 may play important positive roles in the regulation of endothelial cell function, which is a prerequisite step in the process of angiogenesis (Frank et al., 2003). However, previous studies reported that the serum CAV1 protein level was lower in patients with MMD than in controls (Bang et al., 2016a; Chung et al., 2018). In addition, an *in vitro* analysis showed that downregulation of CAV1 suppressed angiogenesis in endothelial cells and induced apoptosis in smooth muscle cells (Chung et al., 2018). Therefore, further studies are needed to investigate the mechanisms of CAV1 and CAVIN2 in the intracranial arteries of MMD.

Similarly, our data also revealed that the GO terms involved in mitochondrial function and the KEGG pathway of oxidative phosphorylation were significantly downregulated in patients with MMD compared with controls. This result was consistent with a previous study, in which data obtained from the MCA of MMD *via* an RNA microarray (Kanamori et al., 2021). Mitochondria and the oxidative phosphorylation system are the pivotal mechanisms of ATP synthesis in maintaining cellular metabolism (Vercellino and Sazanov, 2021). Impaired mitochondrial function plays a central role in several diseases, such as cerebrovascular diseases, hypertension, and neurodegenerative disorders (Sure et al., 2018; Zhou et al., 2021; Rey et al., 2022). Recently, a study has been designed to investigate the relationship between mitochondrial function and MMD. It was found that colony-forming endothelial cells from patients with MMD not only displayed a disrupted mitochondrial morphology, including a shorter and more circular shape, but also aberrant mitochondrial function, manifested as a decrease in the oxygen consumption rate and an increase in intracellular Ca^2+^ concentration (Choi et al., 2018). In addition, Wang et al. (2021) demonstrated that exosomes of hemorrhagic MMD could promote vascular endothelial cell proliferation through the induction of mitochondrial dysfunction and dysregulation of oxidative phosphorylation. Our observations, together with previous results, suggest that functional abnormalities of mitochondria and oxidative phosphorylation may play an important role in the pathogenesis of MMD.

Apart from the findings similar to those of previous research, our study also observed some new insights from the RNA-seq in MCA samples of MMD. Our results showed that the gene expression profile between men and women with MMD was distinctly different. According to the epidemiology results of MMD, there were several distinct differences in the disease pattern according to gender. For instance, it is well-known that MMD occurs more frequently among women, even the incidence of sex ratio (women/men) is shown to be from 1.8 to 2.2 (Baba et al., 2008; Kuriyama et al., 2008; Kainth et al., 2013; Ahn et al., 2014). Additionally, the percentage of ischemia in women is significantly lower than that in men (53.0% vs. 65.9%) (Baba et al., 2008). The age distribution pattern in patients with MMD has changed from 2 peaks to 3 peaks (aged 10–14, 35–39, and 55–59 years) in men, whereas it is still in two peaks (aged 20–24 and 50–54 years) in women (Kuriyama et al., 2008). Taken together, these results suggest that there are sex differences between men and women with MMD. However, the molecular mechanism underlying sex differences in MMD remains clearly unknown. Although previous studies reported that estrogen might be involved in MMD (Levine et al., 1991; Busey et al., 2014; Surmak et al., 2020), all women are postmenopausal in the present study. Thus, we went on to compare the transcriptomic difference between men and women with MMD.

Excitingly, we observed the existence of sex differences in the gene expression in the intracranial samples from patients with MMD *via* the RNA-seq. Our results showed that the gene expression of AQP4 was significantly increased (*p* = 5.81E-05) in men with MMD. AQP4, a major water channel in the brain, plays an important role in the integrity and function of blood–brain barrier (BBB) by reducing inflammatory responses and attenuating the release of pro-inflammatory cytokines (Zhao et al., 2018). Previous studies reported that the level of AQP4 was higher in patients who showed neurological improvement after the onset of ischemic stroke (Ramiro et al., 2020). This suggests that AQP4 may have a neuroprotective role in cerebrovascular disease. Interestingly, Morrison and Filosa (2016) revealed a significant sex difference in AQP4 polarity in contralateral and ipsilateral distal brain regions after MCA occlusion in male but not in female mice. In addition, Solarz et al. (2021) also found pronounced sex differences in BBB permeability in an early-life stress rat model, in which juvenile females showed lower BBB permeability and AQP4 level than males in the dorsal striatum.

For women with MMD, we also observed several higher expressed sex-specific genes, such as SOD3 (*p* = 1.03E-13) and NR4A1 (*p* = 8.16E-10). Extracellular SOD3 is an enzyme that scavenges reactive oxygen species and has been shown to facilitate vascular endothelial function *via* VEGF signaling and anti-inflammatory (Mathias et al., 2021). A previous study found that a higher frailty index was related to a greater improvement in endothelium-dependent dilation with the superoxide scavenger SOD3 in the MCA for old female mice than for old male mice (Cole et al., 2021). NR4A1, a member of the nuclear receptor superfamily, plays an integral role in a plethora of cellular processes, including survival, apoptosis, mitophagy, immunity, and inflammation in response to diverse stimuli (Lin et al., 2004; Rodríguez-Calvo et al., 2017). A previous study has reported that sex steroid hormones, such as estrogens and testosterone, could modulate NR4A1 expression in men and women (Pérez-Sieira et al., 2014). These results suggest the hypothesis that sex-specific DEGs, such as AQP4, SOD3, and NR4A1, in men and women may contribute to sex differences in patients with MMD.

According to the GO analysis, in men with MMD, many sex-specific DEGs take part in the bioprocess of DNA methylation. A previous study has reported that hypomethylation of a specific promoter CpG site of sortilin 1 in endothelial colony-forming cells from MMD was related to endothelial cell function, implying the involvement of hypomethylation of sortilin 1 in MMD pathogenesis (Sung et al., 2018) Recently, a study reported that men with lung cancer showed extensive autosomal DNA hypomethylation and a significantly increased risk of death (Willis-Owen et al., 2021). This result indicates that the level of aberrant DNA methylation in men and women may lead to a different pathophysiology of MMD.

Our study revealed, for the first time, sex differences in the gene expression in the MCA specimens from MMD, which were not reported in previous microarray studies due to over 90% of women in their groups (Okami et al., 2015; Kanamori et al., 2021). Moreover, an understanding of the molecular mechanisms behind sex differences in MMD can lead to new insights into sex-specific pathophysiology and treatment opportunities. Therefore, taking sex differences into account, more research should be conducted to evaluate the molecular mechanisms underlying men and women with MMD in the future.

## Limitations

Nevertheless, it should be noted that the present study has some limitations. First, the relatively small sample size in our RNA-seq experiment may exert some influence on the robustness of our results. Second, adult patients (mean age was 56 years) with MMD were investigated and all women enrolled in our study were postmenopausal women. Thus, a better stratification of the cohort based on age is needed to evaluate our results in premenopausal women and in pediatric MMD. Third, we should consider ethnic differences in MMD, as mentioned above. In the present study, all participants were Chinese, and the RNF213 p.R4810K mutation was not detected in any participant. This is quite different from a similar study previously conducted in Japan, where the rate of this mutation has been reported to be 72.7% in MMD and 11.1% in control patients (Kanamori et al., 2021). Therefore, it is not clear that our findings in this study can be generalized to other ethnic populations. Finally, similar to most bioinformatic research, we merely performed transcriptomic profiling of MMD without any functional investigation. As a consequence, further experimental validation should be conducted to assess the exact biological processes of the genes dysregulated in the development of MMD.

## Conclusion

This transcriptome-wide analysis highlighted the upregulation of genes associated with the organization of ECM and the downregulation of genes involved in mitochondrial function and oxidative phosphorylation in the intracranial arteries of patients with MMD compared with AS-ICASO. In addition, we also displayed, for the first time, the sex dimorphism of gene expression in MCA samples in men and women with MMD, suggesting that sex differences should be taken into account in future studies. In a word, these findings may provide important new insights for further studies to clarify the pathophysiology of MMD.

## Data Availability Statement

The data presented in the study are deposited in the Genome Sequence Archive for Human in BIG Data Center, Beijing Institute of Genomics (BIG), Chinese Academy of Sciences (https://ngdc.cncb.ac.cn/gsa-human/browse/HRA002011), accession number HRA002011.

## Ethics Statement

The studies involving human participants were reviewed and approved by the Institutional Ethics Committee of Zhongnan Hospital, Wuhan University. The patients/participants provided their written informed consent to participate in this study. Written informed consent was obtained from the individual(s) for the publication of any potentially identifiable images or data included in this article.

## Author Contributions

SX, WW, JZ, XL, and JC conceived and designed experiments. SX, WW, TC, LD, JS, XW, TZ, and ZL performed experiments. SX, WW, and FZ analyzed data, interpreted experimental results, prepared figures, and drafted this manuscript. SX, WW, FZ, TC, LD, JS, XW, TZ, ZL, JZ, XL, and JC edited and revised this manuscript and approved the final version of this manuscript. All authors contributed to the article and approved the submitted version.

## Funding

This work was supported by the National Natural Science Foundation of China (NSFC 81771280 and 82171326) and by the Research Fund from Medical Sci-Tech Innovation Platform of Zhongnan Hospital, Wuhan University(PTXM2021006).

## Conflict of Interest

The authors declare that the research was conducted in the absence of any commercial or financial relationships that could be construed as a potential conflict of interest.

## Publisher's Note

All claims expressed in this article are solely those of the authors and do not necessarily represent those of their affiliated organizations, or those of the publisher, the editors and the reviewers. Any product that may be evaluated in this article, or claim that may be made by its manufacturer, is not guaranteed or endorsed by the publisher.
