# Supplementary material for: Transcriptomic Profiling of Intracranial Arteries in Adult Patients With Moyamoya Disease Reveals Novel Insights Into Its Pathogenesis
Source: Front Mol Neurosci. 2022 May 31;15:881954. doi: 10.3389/fnmol.2022.881954 (PMC9197469; doi:10.3389/fnmol.2022.881954)
Supplement: Supplementary file 1 [file Table_1.docx]

**Supplemental Table S1. Details of the baseline and clinical characteristics of the RNA-Seq patients.**

| **No. of Patients** | **Age (years)** | **Sex** | **Post-menopause** | **Lesion location** | **Suzuki stage** | **Initial symptom** | **Medical history** | **Mutation of RNF213** | **Interval time (day)** |
| --- | --- | --- | --- | --- | --- | --- | --- | --- | --- |
| MMD1 | 65 | F | Yes | L-lateral | R0/L3 | CI | NA | No | 99 |
| MMD2 | 56 | F | Yes | Bilateral | R3/L3 | ICH | HT | No | 110 |
| MMD3 | 58 | F | Yes | Bilateral | R2/L3 | TIA | HT | No | 106 |
| MMD4 | 58 | F | Yes | Bilateral | R3/L3 | IVH | NA | No | 152 |
| MMD5 | 50 | F | Yes | L-lateral | R0/L2 | CI | NA | No | 232 |
| MMD6 | 52 | F | Yes | R-lateral | R3/L0 | ICH | DM | No | 109 |
| MMD7 | 51 | F | Yes | Bilateral | R2/L3 | TIA | DM | No | 97 |
| MMD8 | 56 | F | Yes | Bilateral | R5/L4 | IVH | NA | No | 121 |
| MMD9 | 60 | M | NA | Bilateral | R3/L3 | ICH | NA | No | 96 |
| MMD10 | 48 | M | NA | Bilateral | R3/L3 | CI | NA | No | 110 |
| MMD11 | 57 | M | NA | L-lateral | R0/L3 | ICH | NA | No | 135 |
| MMD12 | 52 | M | NA | Bilateral | R3/L4 | CI | NA | No | 108 |
| MMD13 | 66 | M | NA | Bilateral | R3/L3 | CI | NA | No | 100 |
| MMD14 | 49 | M | NA | L-lateral | R0/L5 | SAH | NA | No | 16 |
| MMD15 | 52 | M | NA | Bilateral | R2/L4 | CI | HT | No | 96 |
| MMD16 | 49 | M | NA | Bilateral | R3/L2 | CI | HT | No | 243 |
| AS-ICASO1 | 51 | M | NA | L-MCA | NA | CI | HT | No | 103 |
| AS-ICASO2 | 58 | M | NA | L-ICA | NA | TIA | HL | No | 104 |
| AS-ICASO3 | 48 | M | NA | L-MCA | NA | CI | HT | No | 98 |
| AS-ICASO4 | 72 | F | Yes | R-MCA | NA | CI | HT+DM | No | 110 |
| AS-ICASO5 | 69 | F | Yes | L-ICA | NA | CI | HT+DM | No | 106 |

MMD, moyamoya disease; AS-ICASO, atherosclerosis associated intracranial artery stenosis/occlusion. F, female; M, male; L, left; R, right. MCA, middle cerebral artery; ICA, internal carotid artery. TIA, transient ischemic attack; CI, cerebral infarction; IVH, intraventricular hemorrhage; ICH, intracerebral hemorrhage; SAH, subarachnoid hemorrhage. HT, hypertension; HL, hyperlipidemia; DM, diabetes mellitus. Mutation of RNF213 indicates p.R4810K mutation. NA, no association. Interval time indicates the duration from the last symptom until the surgery.
