# Supplementary material for: Transcriptomic Profiling of Intracranial Arteries in Adult Patients With Moyamoya Disease Reveals Novel Insights Into Its Pathogenesis
Source: Front Mol Neurosci. 2022 May 31;15:881954. doi: 10.3389/fnmol.2022.881954 (PMC9197469; doi:10.3389/fnmol.2022.881954)
Supplement: Supplementary file 2 [file Table_2.docx]

**Supplemental Table S2. The list of primer sequences used in this study.**

| **Gene** | **Forward primer (5’-3’)** | **Reverse primer (5’-3’)** |
| --- | --- | --- |
| GAPDH | GGAGCGAGATCCCTCCAAAAT | GGCTGTTGTCATACTTCTCATGG |
| TIMP1 | ATCCATCCCCTGCAAACTGC | GGCAGGATTCAGGCTATCTGG |
| CAVIN2 | TGCCTAGCAGTGAGCAGATG | CGCGTAGCTACCCTCATAGC |
| MT-CO2 | ACTGAACCTACGAGTACACCGA | TTAATTCTAGGACGATGGGCATG |
| RNF213 | TACTGGGTGGTCTTCCCTTCTC | TGTTCCCCTATGCAGTGATCC |

GAPDH, glyceraldehyde 3-phosphate dehydrogenase; TIMP1, metallopeptidase inhibitor 1; CAVIN2, caveolae associated protein 2; MT-CO2, mitochondrially encoded cytochrome c oxidase II; RNF213, ring finger protein 213.
