# Supplementary figures and images for: Transcriptomic Profiling of Intracranial Arteries in Adult Patients With Moyamoya Disease Reveals Novel Insights Into Its Pathogenesis
Source: Front Mol Neurosci. 2022 May 31;15:881954. doi: 10.3389/fnmol.2022.881954 (PMC9197469; doi:10.3389/fnmol.2022.881954)

**Figure S1. Heatmap for hemorrhagic and ischemic MMD**

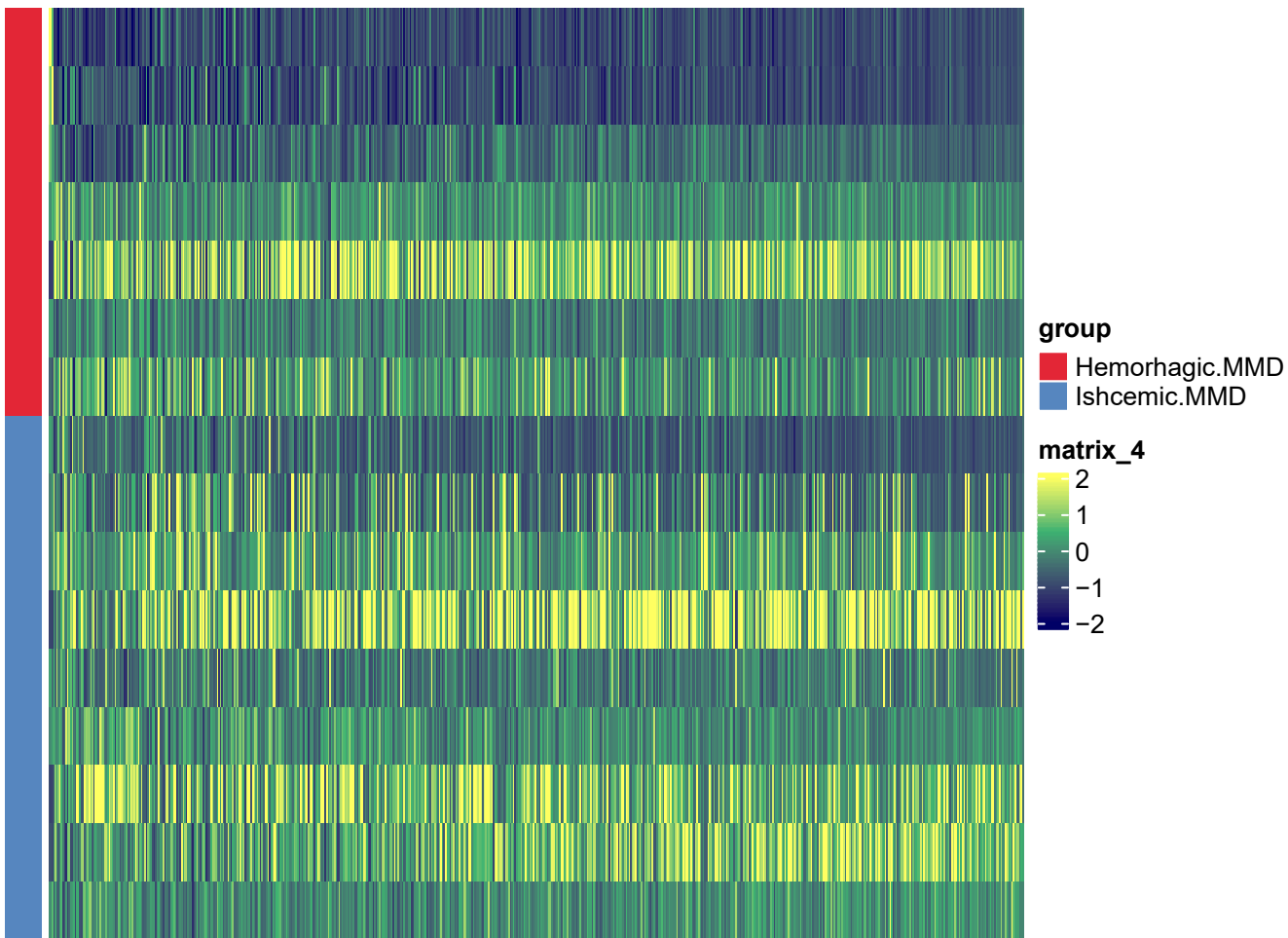

Supplement: Supplementary file 5 [file Image_1.pdf]

**Figure S2. PCA for hemorrhagic and ischemic MMD**

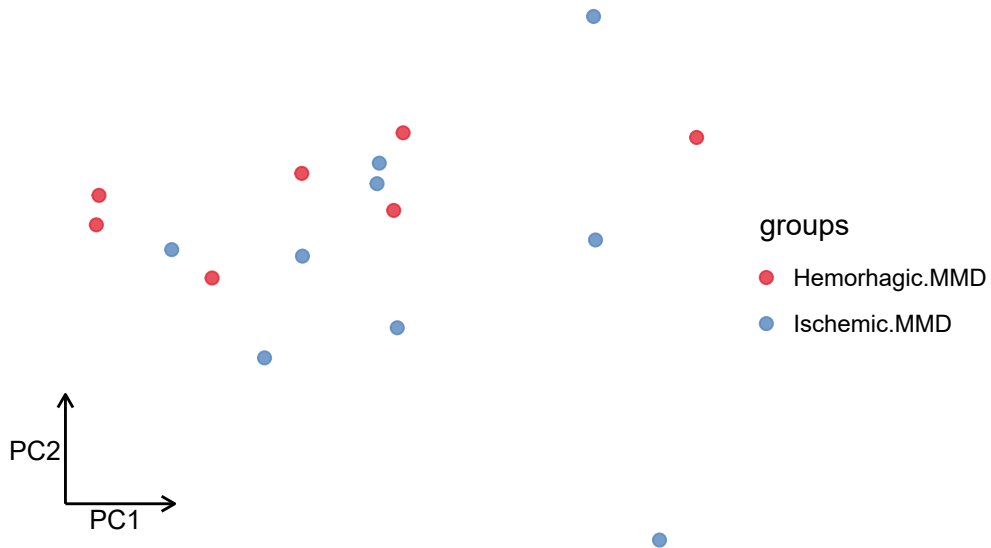

Supplement: Supplementary file 6 [file Image_2.pdf]
